# A Unique Case of Acute Respiratory Distress Syndrome Secondary to Rheumatoid Lung Disease With Administration of Anti-Tumor Necrosis Factor Alpha (TNFα) Agent

**DOI:** 10.7759/cureus.37990

**Published:** 2023-04-22

**Authors:** Daniel Sherlock, Hardeep S Ahdi, Raju Mehta

**Affiliations:** 1 Internal Medicine, Advocate Lutheran General Hospital, Park Ridge, USA; 2 Critical Care Medicine, Advocate Lutheran General Hospital, Park Ridge, USA

**Keywords:** acute respiratory distress syndrome (ards), rheumatoid granulomatous disease, rheumatoid lung disease, rheumatoid arthriitis, adalimumab (humira)

## Abstract

Patients with rheumatoid arthritis (RA) may experience complications directly from the disease process or from immune-modulating agents used to treat RA. Adalimumab is a recombinant human monoclonal antibody directed against tumor necrosis factor alpha (TNFα) which has been increasingly used in the management of inflammatory and autoimmune diseases. Acute lung injury has been associated with the use of anti-TNFα agents, but the association with adalimumab is rare. Here we present a case of a patient with RA-associated lung disease who developed acute respiratory distress syndrome while being treated with adalimumab. Adalimumab-related lung injury is less common than lung injury associated with other anti-TNFα drugs, thus clinicians should be aware of this condition, as prompt recognition and supportive management can help prevent worsening outcomes.

## Introduction

Pulmonary manifestations of rheumatoid arthritis (RA) have been well documented in the literature, the majority of which typically occur within five years of diagnosis of rheumatoid joint disease [1]. There are several reports of anti-tumor necrosis factor alpha (TNFα)-induced lung disease, most often secondary to etanercept, especially in patients with rheumatologic diseases [2,3]. Few case reports have associated adalimumab with interstitial pneumonia. Pathological findings associated with adalimumab-related lung injury include ground glass opacities, honeycombing, and reticulonodular opacities [4]. This case offers a fascinating insight into a patient who had inflammatory granulomatous lung disease treated with adalimumab and who experienced subsequent lung injury. While the exact mechanisms associated with anti-TNFα related lung injury are unknown, it is hypothesized that inhibition of TNFα may promote a profibrotic Th2 response in the lungs [5,6]. The best response to this type of reaction appears to be discontinuing the responsible agent and providing supportive care.

## Case presentation

**Figure 1:**
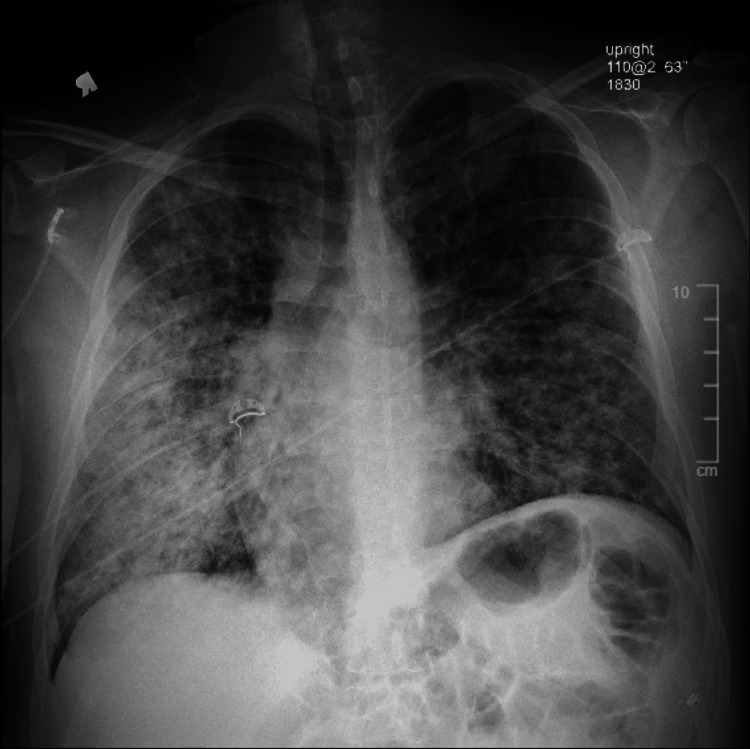
Chest X-ray on Admission

A 35-year-old male with a history of seropositive RA and endobronchial ultrasound-guided fine needle aspiration showing evidence of granulomatous disease presented for evaluation of fever, cough, and shortness of breath. The patient was suspected to have interstitial lung disease (ILD) from underlying RA, and had been taking adalimumab every two weeks for several months. Tuberculosis testing was negative. On arrival at the hospital, he was febrile, tachycardic, tachypneic, and hypoxic. He was transferred to the medical intensive care unit for acute hypoxic respiratory failure. Rapid coronavirus disease 2019 (COVID-19) testing was negative. Labs revealed a white blood cell count of 16.7 K/mcL, procalcitonin 0.56 ng/mL, D-dimer 1.23 mg/L (FEU), erythrocyte sedimentation rate 116 mm/hr, C-reactive protein 37 mg/dL, rheumatoid factor 113.5 IU/mL, and ferritin 2176 ng/mL. Anti-cyclic-citrullinated antibodies were positive. Transthoracic echocardiogram (TTE) showed no regional wall motion abnormalities, a left ventricular ejection fraction of 75%, and dilated inferior vena cava, but was otherwise unremarkable. Chest x-ray showed diffuse pulmonary opacities (Figure 1).

**Figure 2:**
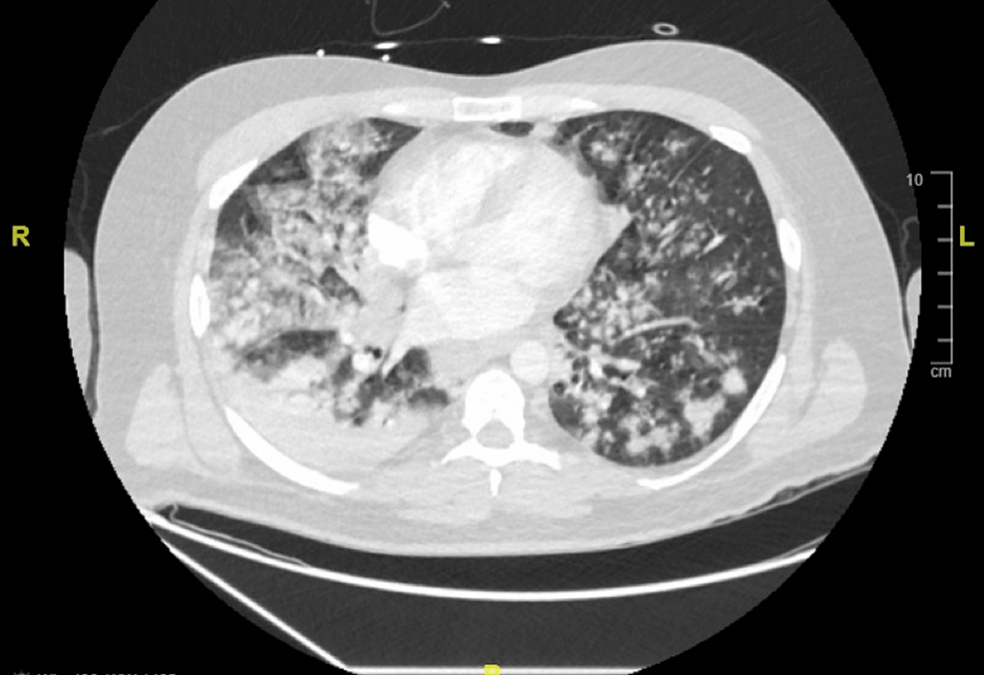
CT Chest on Admission

CT chest showed extensive bilateral consolidations with nodular opacities, and no pulmonary embolism (Figure 2).

A diagnosis of severe acute respiratory distress syndrome (ARDS) was made based on the acute onset, bilateral lung infiltrates seen on chest radiography, and arterial oxygen pressure (PaO2)/fraction of inspired oxygen (FiO2) ratio of less than 100 mmHg. He was treated with broad-spectrum antibiotics and antifungals in the setting of potential atypical and opportunistic infections. His hospital course was complicated by ARDS requiring lung protective mechanical ventilation, chemical paralysis, and prone positioning. A bronchoscopy was performed with bronchoalveolar lavage positive for very rare coagulase-negative staphylococci (CoNS) and *Enterococcus faecium*, and negative for fungal or acid-fast bacteria cultures. There were no signs of diffuse alveolar hemorrhage. The patient was started on high-dose glucocorticoids and received one dose of rituximab. Trimethoprim-sulfamethoxazole was started for *Pneumocystis jirovecii *pneumonia prophylaxis and acyclovir for viral prophylaxis while on rituximab and steroids. He was liberated from the ventilator and his oxygen requirements continued to improve over time.

## Discussion

RA is an autoimmune and inflammatory disease process that most often affects the joints, but can also affect other tissues throughout the body including the eyes, heart, and lungs. Rheumatoid lung disease is characterized by inflammation of the lining of the lungs and can lead to the development of ILD, pleural thickening, and pleural effusions. The underlying mechanisms behind airway and parenchymal damage have not been clearly defined [7]. However, the combination of direct injury to lung tissue along with factors such as oxidative stress, hypoxia, and immune function seems to play a role in disease development. Treatment of rheumatoid lung disease occurs in a stepwise approach and includes traditional disease-modifying anti-rheumatic drugs (DMARDs), biologic agents, glucocorticoids, and tofacitinib [8].

Recently, biologic agents specifically have started to play a larger role in the treatment of RA. TNF-α antagonists such as etanercept, infliximab, adalimumab, certolizumab pegol, and golimumab have been approved for use in the treatment of RA and show very promising results. These drugs act on the inflammatory and immune response pathway activated by TNF, which is made intracellularly, most often by activated macrophages. TNF can then go on to activate signaling pathways that lead to the release of cytokines and the initiation of apoptosis [9]. By inhibiting this pathway, anti-TNFα drugs can reduce inflammation. These drugs are generally well tolerated with the most common side effects being headaches, rashes, cough, nausea, vomiting, and diarrhea. However, more serious side effects have been reported including infections, increased risk of malignancies, drug-induced lupus, and acute lung injury [9]. These agents have also been shown to increase the risk of developing other autoimmune diseases or reactivating infections, sarcoidosis, or other interstitial lung diseases [10].

Anti-TNF-related ILD has a prevalence of 0.5-3%, and acute lung injury has been reported most often with the use of etanercept, although there are several cases related to adalimumab use [11]. The first case of adalimumab-induced ILD occurred in 2014 in Brazil [12]. Among anti-TNF drugs, adalimumab has the least reported lung toxicity. Most reported cases of adalimumab-induced lung injury have an onset of two to six months, although shorter onsets have also been reported [4]. In this case, the onset was around 2.5 months from the patient's first adalimumab treatment. It is not clear why this last administration of adalimumab led to the patient developing ILD. The exact mechanism by which adalimumab causes acute lung injury is not well understood, but it is theorized that it may be related to an imbalance between pro-and anti-inflammatory cytokines. There are also other cases reported that seem to be related to an underlying infection, such as viral illness, or a triggering event. The patient in this case worked in the postal service and may have been exposed to an environmental trigger as his infectious work-up was unremarkable. Acute lung injury in patients taking adalimumab is rare and is typically seen in patients with other underlying risk factors for lung disease. Our patient's greatest risk factor was his underlying RA granulomatous lung disease. Symptoms can present as dyspnea, fatigue, cough, and fever. Typical findings on CT show ground glass opacities, honeycombing, and reticulonodular opacities. [13]

The treatment of adalimumab-induced ILD consists of discontinuation of the drug in combination with supportive therapies such as oxygen supplementation and steroids. Usually, patients begin to see improvement within one to two weeks of stopping adalimumab. In 65% of cases, patients will make a full recovery, but the failure of treatment often results in death [13].

## Conclusions

The use of anti-TNFa medications in patients with rheumatological diseases has been increasing over the last few years, which will potentially shed some insight into additional unwanted side effects. While it is rare, lung injury, which can potentially be fatal, has been recognized in all available anti-TNF agents. It is often easy to consider the beneficial effects of these new biologic agents without considering their potentially harmful side effects. Prompt recognition of symptoms and discontinuation of the offending agent seems to be the best treatment option. Extra consideration should be taken for any patient with a history of lung diseases such as rheumatoid lung disease, interstitial lung disease, or other known lung insult. Clinicians should be more aware of these potentially dangerous side effects when creating treatment plans and discussing options with patients and patients should seek medical attention promptly if they experience shortness of breath or other worrying symptoms.
